# A Rare Case of Psychosis Comorbid with Acromegaly Secondary to Pituitary Macroadenoma: A Case Report

**DOI:** 10.1192/j.eurpsy.2026.12170

**Published:** 2026-07-31

**Authors:** B. Gokkaya, N. P. Basaran Arslan, F. B. Atak Akkus, H. Kaya, E. Goka

**Affiliations:** Psychiatry, Ankara Bilkent City Hospital, Ankara, Türkiye

## Abstract

**Introduction:**

Acromegaly results from chronic overproduction of growth hormone (GH), most frequently due to a pituitary adenoma. Symptoms arise from elevated hormone levels or the tumor’s mass effect itself, leading to acral tissue overgrowth and neurological or visual deficits. Neuropsychiatric symptoms are in a wide range and not well understood.

**Objectives:**

We describe an uncommon case in which psychosis developed in association with acromegaly caused by a GH-secreting pituitary macroadenoma. By presenting this case, we aim to discuss potential links between the two conditions and contribute to the understanding of this unusual clinical overlap.

**Methods:**

A 45-year-old woman was admitted with psychotic symptoms concurrent with an acromegaly diagnosis. Comprehensive assessments included psychiatric evaluation using the Positive and Negative Syndrome Scale (PANSS), also brain and pituitary Magnetic Resonance Imaging (MRI), and laboratory tests. Psychosis was treated with antipsychotics. Endocrine therapy was also initiated in parallel with antipsychotics.

**Results:**

The patient exhibited anger, agitation, and aggression in the emergency service. Physical examination revealed prognathism, macroglossia, and coarse facial features typical of acromegaly. Psychiatrically, she showed disorganized thought and persecutory, referential, and somatic delusions, interpreting bodily changes delusionally. Her psychiatric symptoms began in her 30s, coinciding with the discovery of a headache and a pituitary mass. Over the years, she developed persistent delusions with mystical and persecutory themes and functional decline. The patient exhibited progressive decline in speech and cognitive functions. The MRI showed a 23x28x32 mm lobulated sellar mass, heterogeneously isointense on T1- and T2-weighted images, enhancing with contrast, consistent with a macroadenoma. The lesion invaded the cavernous sinuses, encased the internal carotid arteries, obliterated the suprasellar cistern, compressed the hypothalamus and optic chiasm, and displaced cerebral arteries. (image 1, image 2) Lab tests revealed elevated Insulin Like Growth Factor 1 (IGF-1) and GH levels.

Psychotic symptoms significantly improved following treatment with clozapine (300 mg/day), aripiprazole (30 mg/day), and lanreotide (90 mg monthly). This demonstrates the benefits of combining psychiatric and endocrinological treatment in cases of psychosis with pituitary adenoma.

**Image 1:**
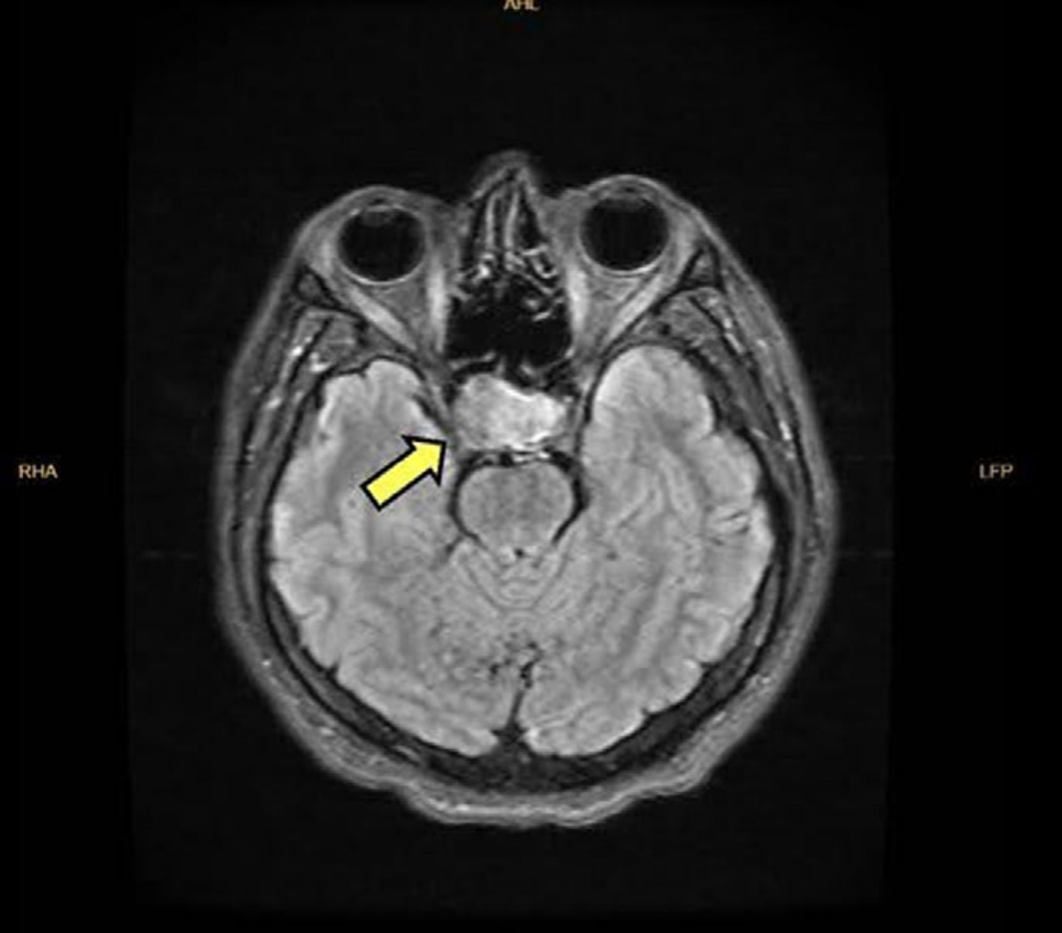

**Image 2:**
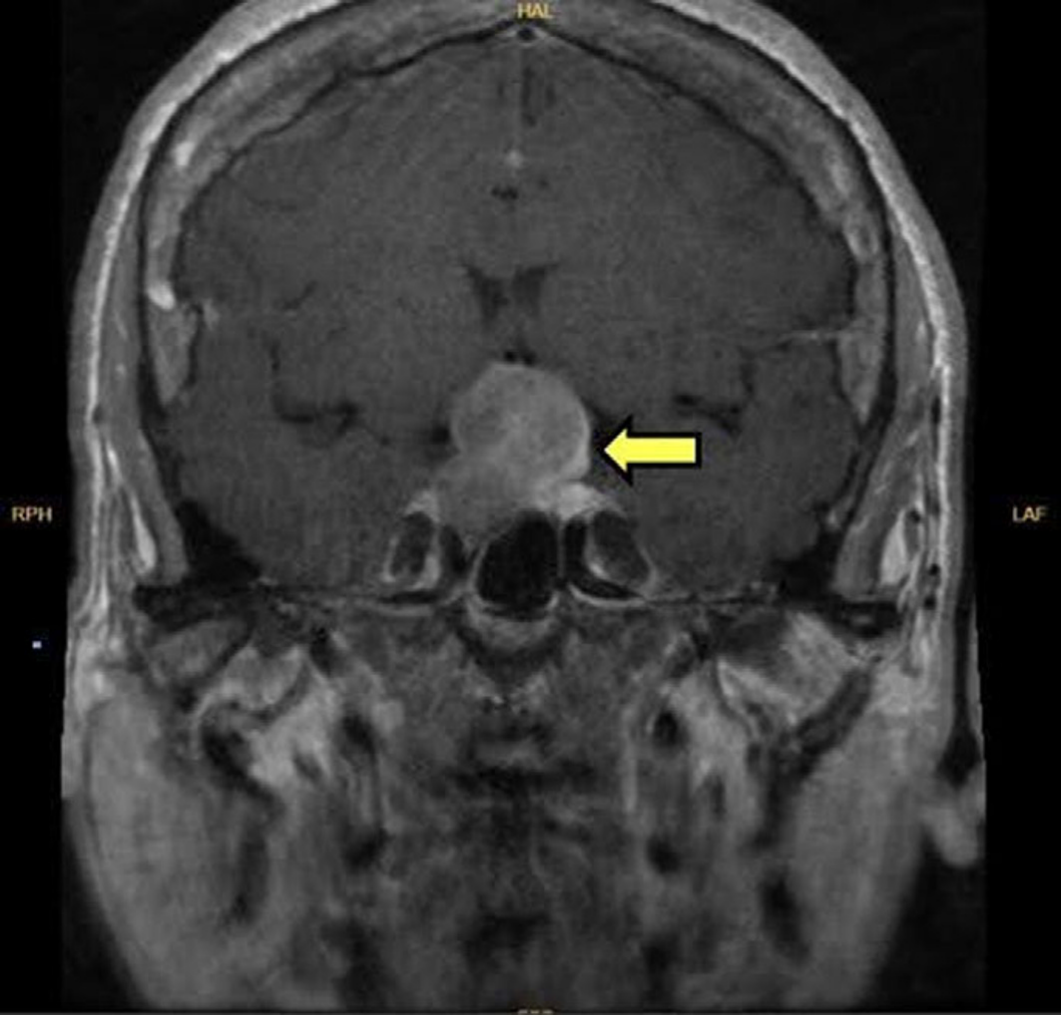

**Conclusions:**

While psychosis comorbid with acromegaly is rarely reported, a causal relationship remains unclear. Possible mechanisms include tumor-induced structural, functional, or hormonal brain changes. Case reports like this may provide insight into the neuropsychiatric impact of pituitary adenomas and highlight the need for multidisciplinary management.

**Disclosure of Interest:**

None Declared

